# Infection-interactions in Ethiopian village chickens

**DOI:** 10.1016/j.prevetmed.2014.07.002

**Published:** 2014-11-15

**Authors:** J.M. Bettridge, S.E. Lynch, M.C. Brena, K. Melese, T. Dessie, Z.G. Terfa, T.T. Desta, S. Rushton, O. Hanotte, P. Kaiser, P. Wigley, R.M. Christley

**Affiliations:** a Institute of Infection and Global Health, University of Liverpool, Leahurst Campus, Liverpool CH64 7TE, United Kingdom; b International Livestock Research Institute, Addis Ababa, Ethiopia; c Debre Zeit Agricultural Research Centre, Ethiopian Institute for Agriculture Research, Debre Zeit, Ethiopia; d Centre for Genetics and Genomics, School of Biology, University of Nottingham, University Park, Nottingham NG7 2RD, United Kingdom; e School of Biology, Newcastle University, Newcastle Upon Tyne, NE1 7RU, United Kingdom; f The Roslin Institute and Royal (Dick) School of Veterinary Science, University of Edinburgh, Midlothian EH25 9RG, United Kingdom; g NIHR Health Protection Research Unit in Emerging and Zoonotic Infections, Liverpool, L69 7BE, United Kingdom

**Keywords:** Co-infection, Host-parasite interaction, Constrained ordination, Village chicken, Ethiopia

## Abstract

Chickens raised under village production systems are exposed to a wide variety of pathogens, and current or previous infections may affect their susceptibility to further infections with another parasite, and/or can alter the manifestation of each infection. It is possible that co-infections may be as important as environmental risk factors. However, in cross-sectional studies, where the timing of infection is unknown, apparent associations between infections may be observed due to parasites sharing common risk factors. This study measured antibody titres to 3 viral (Newcastle disease, Marek's disease and infectious bursal disease) and 2 bacterial (*Pasteurella multocida* and *Salmonella*) diseases, and the infection prevalence of 3 families of endo- and ecto-parasites (*Ascaridida*, *Eimeria* and lice) in 1056 village chickens from two geographically distinct populations in Ethiopia. Samples were collected during 4 cross-sectional surveys, each approximately 6 months apart. Constrained ordination, a technique for analysis of ecological community data, was used to explore this complex dataset and enabled potential relationships to be uncovered and tested despite the different measurements used for the different parasites. It was found that only a small proportion of variation in the data could be explained by the risk factors measured. Very few birds (9/1280) were found to be seropositive to Newcastle disease. Positive relationships were identified between *Pasteurella* and *Salmonella* titres; and between Marek's disease and parasitic infections, and these two groups of diseases were correlated with females and males, respectively. This may suggest differences in the way that the immune systems of male and female chickens interact with these parasites. In conclusion, we find that a number of infectious pathogens and their interactions are likely to impact village chicken health and production. Control of these infections is likely to be of importance in future development planning.

## 1 Introduction

Poultry kept in village production systems are exposed to a wide range of potential pathogens, yet most epidemiology studies have tended to focus on single infections. The importance of interactions between pathogens has been shown in humans (Pullan and Brooker, 2008, Nacher, 2011), wildlife (Jolles et al., 2008, Telfer et al., 2008) and extensively farmed livestock species (Thumbi et al., 2014). However, determining the direction and strength of any interactions remains a complex issue.

There is widespread recognition of the importance of village poultry in developing countries (Copland and Alders, 2005, Mack et al., 2005). Poultry are easily accessible, even to the poorest households or those with a lack of able-bodied workers, as they require minimal land, labour or financial inputs. They can scavenge for food, and do not compete for food resources with humans. They are normally in close proximity to the household, enabling them to be managed by women and children while placing few additional burdens on these groups, as they may be kept under minimal supervision. This role in managing a household asset gives women greater control over the income from sale of poultry and eggs, and this can be used to support child education. As such, the improvement programs for family poultry have the potential to contribute to several of the UN's Millennium Development Goals (Alders and Pym, 2009).

Infectious disease is recognised as one of the major constraints to developing backyard poultry production (Guèye, 1998, Mack et al., 2005). Control of Newcastle disease virus (NDV) has been identified as the most critical intervention in numerous studies throughout Africa, and other interventions such as improving feed, housing or controlling parasites are only effective if used in conjunction with vaccination (Dwinger and Unger, 2006). A number of seroprevalence surveys have demonstrated exposure to NDV in several areas of Ethiopia (Tadesse et al., 2005, Mazengia et al., 2010, Chaka et al., 2012), although little attention has been paid as yet to control strategies. However, NDV is not the only problem; chickens under a village production system are exposed concurrently or consecutively to a number of different pathogens. In Ethiopia seroprevalence surveys in village chickens have identified the presence of infectious bursal disease (Mazengia et al., 2010, Chaka et al., 2012, Jenbreie et al., 2012), salmonellosis (Berhe et al., 2012, Alebachew and Mekonnen, 2013), pasteurellosis and mycoplasma infection (Chaka et al., 2012). Marek's disease has also been identified in village chickens kept under intensive management (Duguma et al., 2005). Parasitic diseases, including coccidiosis (Ashenafi et al., 2004, Luu et al., 2013), helminths (Tolossa et al., 2009, Molla et al., 2012) and ectoparasites (Belihu et al., 2009, Tolossa et al., 2009) have also been demonstrated to be highly prevalent in the country.

Host survival may be severely affected by multiple, coincident infections (Jolles et al., 2008) and there is evidence from studies of wild rodents that existing infections may pose greater risks for further infections than environmental variables (Telfer et al., 2010). Associations between diseases may be the result of direct interactions between the pathogens themselves or indirectly, via the bird's immune system, such that their impact on the host may be altered. Pre-infection with Marek's disease has been observed to alter the clearance of certain *Eimeria* species (Biggs et al., 1968), while both *P. multocida* and *Salmonella enterica* serovar Enteritidis infections have been shown to be more severe in the presence of pre-infection with the nematode *Ascaridia galli* (Dahl et al., 2002, Eigaard et al., 2006). Many of these interactions are thought to be mediated through the altered differentiation of T-lymphocytes; whereas protective cell-mediated responses to intracellular microbial pathogens are primarily driven by T helper 1 (Th1) cells, protection to extracellular infections, including macroparasites, usually requires antibody and primes the immune system towards a Th2-type response. Hosts in a natural environment may have limited ability to effectively mount both types of immune response simultaneously, particularly where they are constrained by limited resources (Jolles et al., 2008). The helminth *Ascaridia galli* has also been shown to reduce the antibody response to vaccination with NDV (Horning et al., 2003), therefore determining pathogen interactions may have implications for disease control programmes. Indeed, the health consequences of multiple low-grade infections may become more significant as interventions reduce the impact of single diseases (Pullan and Brooker, 2008).

However, apparent interactions between infections may arise simply because diseases have other risk factors in common, such as similar transmission routes, temporal patterns of exposure, or host factors, such as sex, age, socio-economic status or behaviours (Hellard et al., 2012). The use of generalised linear mixed models, as proposed by Fenton et al. (2010) for the analysis of macroparasite data, can account for such factors, but may be less useful for serological surveys which measure the host's adaptive immune response rather than the pathogen itself. Serological data present the added difficulty that they do not necessarily represent a current infection; the order of infection is important in determining the immune response to each single infection, and it is thought that susceptibility to new co-infections may return to normal soon after the clearance of the first infection (Telfer et al., 2010). Therefore models which assume a directional relationship are unsuited to cross-sectional serological data (Hellard et al., 2012).

Although there have been several laboratory studies of interactions between pairs of pathogens in poultry, in a village situation pathogens exist in communities, with the possibility of multiple interactions occurring. This study therefore investigated the epidemiology and ecology of co-infection with a range of pathogens in Ethiopian backyard chickens, with the main objectives being (i) to assess patterns of co-infection, and (ii) to identify common risk factors for co-infections. The range of pathogens studied necessitated the collection of both serological and parasitological data, and a method of combining these different infection measures was sought. Ordination methods are commonly used in ecology for community analysis: Here they have been applied to this community of pathogens, where each bird may be thought of as a “site”, which has the potential to be exploited by any of the micro- and macroparasite species.

Two main techniques were used to explore this data set. Principal component analysis (PCA) is an ordination technique which seeks to extract the main trends in the data set such that it may be explained by a few linearly uncorrelated principal components, which will be less than or equal to the number of original variables. The main structures in the data may then be displayed in a graph(s) constructed from the reduced set of orthogonal axes. Graphs, known as biplots, can be scaled according either to the distances between sites or the correlations between variables (Zuur et al., 2007). Redundancy analysis (RDA) is a canonical ordination technique which combines regression with principal component analysis, and thus tests the relationships of a set of explanatory variables to the multivariate response data. This study highlights the use of both of these techniques to explore infection-interactions in Ethiopian village chickens.

## 2 Materials and methods

This study was approved by the University of Liverpool Committee on Research Ethics (reference RETH000410). The study was carried out between May 2011 and November 2012 in two geographically distinct woredas (administrative districts), Horro and Jarso, within the Oromia region of Ethiopia. Woredas are divided into smaller administrative districts, called kebele, which may comprise several villages. A group of villages which are linked by trading networks is collectively known as a market-shed (Tadelle et al., 2003). Within each region two market–sheds and two kebele per market shed were purposely selected on the basis of their willingness to participate, in consultation with local representatives of the Department of Agriculture and the communities. The eight selected kebele were believed, locally, to be representative of other kebele within the woreda. Each kebele was visited on four occasions; in May/June and October/November in each year of the study. These visits were timed for before and after the main rainy season, which occurs between June and September.

A list of names of all household heads in each kebele, grouped by sub-area, was obtained from the respective kebele agricultural development agents. Systematic random sampling was used to select potential participants by selecting every *n*th name from each list with a starting place chosen at random, to create lists of 108 potential participants for each kebele. The selected names were split into four groups, one for each sampling season, so that different households were selected to sample on each visit to the kebele. Approximately 30% more names than were required were selected to allow for exclusions or non-participation. A total of 640 households were recruited over the course of the study.

Each kebele visit took place over 2–4 days. Ethiopian staff fluent in the local languages and in English were recruited and trained to collect data for this study. The field teams, assisted by local representatives, visited each selected household to take part in a questionnaire survey and for two of their chickens to be sampled. Households which did not own at least two indigenous-breed chickens of over 6 months of age were excluded from the study. Information on management and disease occurrence within the household flock over the previous 12 months was collected in a questionnaire interview in the local language with an adult member of the household, normally either the household head or the main carer for the chickens. In addition, two chickens of at least 6 months of age were randomly selected from the household flock and each underwent clinical examination, was condition scored using a 0–3 grading scale (Gregory and Robins, 1998), and was scored for lice using a timed count of 3 areas of the body – one side of the keel, the back and the rump – and a total count of lice at the base of the flight feathers of one wing and the tail feathers (Clayton and Drown, 2001). Birds were also scored for the degree of hyperkeratosis of the legs and feet (none, mild or severe), and scrapings were taken to confirm the presence of *Cnemidocoptes mutans* (scaly leg mite). Data on the chickens’ age, origin and current status of production was collected from the owner. Blood samples were drawn from the brachial vein of the selected birds into sodium citrate and faecal samples were collected from individual birds wherever possible; environmental faecal samples were taken to represent the household, if individual samples were not forthcoming. All samples were kept chilled and were transported to the laboratory for testing.

Serological testing was carried out for NDV antibodies by haemagglutination inhibition (HAI) assays, according to the OIE (2009), and for other bacterial and viral infections by enzyme-linked immunosorbent assays (ELISA). Infectious bursal disease (IBD) virus antibodies were tested for using a commercial kit (Flockscreen, x-OvO, Dunfermline, UK), while antibody ELISAs were developed in-house for *P. multocida* (PM) and *Salmonella* O9 serotypes, using *S. enterica* serovar Gallinarum (SG) antigen, according to the protocols by Beal et al. (2004); and to Marek's disease virus (MDV) according to the protocol described by Zelnik et al. (2004). The latter assay required that samples were tested against a lysate of both MDV-infected and uninfected chicken kidney cells to identify false positives. This was done using a mixed-effects linear regression model to estimate the expected optical density (OD) against the uninfected lysate after controlling for differences between plates. Those samples with a residual more than 3 standard deviations above the mean were deemed to be cross-reactors and discarded from further analyses. Samples for all ELISA's were tested in triplicate wherever possible, or in duplicate when reagents became limited; this affected all tests, with the exception of *Pasteurella*, and samples from all areas and seasons, with the exception of those from the third sampling season. All samples with high variation between replicates were retested. Positive and negative controls were run on each plate. Optical densities for all ELISA samples were converted into a ratio to the positive control (s:p ratio) to allow comparison of samples tested on different plates, using Eq. (1). Mixed-effects linear regression was used to control for the variation between plates and the bird-level residuals were taken as an estimation of the antibody response.

(1) $$\text{s}:\text{p}\;\text{ratio}=\frac{\text{mean}\;\text{sample}\;\text{OD}-\text{negative}\;\text{control}\;\text{OD}}{\text{positive}\;\text{control}\;\text{OD}-\text{negative}\;\text{control}\;\text{OD}}$$

Faecal samples were examined for *Eimeria* spp. oocysts and nematode eggs using a modified version of the concentration McMaster technique and identified to genus level where possible using published keys (Permin and Hansen, 1998). Only the most common egg types, which could be attributed to nematodes of the Ascaridida order *Ascaridia galli* or *Heterakis gallinarum*, were counted. These eggs were counted as a single group, as it was not possible to accurately differentiate the two species. Although other types of nematode eggs were seen in low numbers, there were no other frequently occurring species.

Analyses were performed using R software (R Development Core Team, 2008) using the vegan package (Oksanen et al., 2011), and according to the methods described by Borcard et al. (2011). A matrix of response variables was constructed from the ELISA s:p ratios for the viral and bacterial pathogens (IBD, PM, SG and MDV); counts of the three parasites (Ascaridida, *Eimeria* and lice) and the hyperkeratosis grading. In order to make all response variables dimensionally homogeneous, each variable was scaled by centring values on the mean and dividing by the standard deviation. This ensured additional weights were not given to the parasites with larger ranges of values and fulfils one of the basic requirements for application of PCA.

In order to test whether the results were features of the data (different data types, presence of outliers), data were re-analysed following a number of prior transformations, including log transformations of the ELISA s:p ratios, square root transformations of the individual parasite count data, and Hellinger transformations of the parasite count matrix. In addition, data were re-analysed following dichotomisation. The IBD ELISA was dichotomised using the manufacturer's recommended cut-off (0.306). The range of s:p ratios to the ELISA tests developed in-house did not demonstrate clear cut-off points, and sufficient negative sera were not available to confidently determine positive cut-offs for these tests. A small panel of sera from birds with known exposure to vaccines or experimental infection were used to determine a conservative cut off value for each test which would still detect all known positives. Cut-off values chosen were 0.34, 0.61 and 0.29 for *Salmonella*, *Pasteurella* and MDV, respectively. The analyses were also run with preselected cut-offs of both 0.3 and 0.5 for these three ELISAs. Presence/absence was used to dichotomise the parasite counts and hyperkeratosis.

Initially, an unconstrained PCA was carried out on the matrix to identify correlations between parasites; a second matrix of explanatory variables, including household data and bird signalment, was then incorporated using RDA. A category “production status” was used to combine data on sex and the current state of production (in lay, not in lay, brooding eggs, rearing chicks or male). Each centred response variable was regressed on the explanatory variables and the fitted values combined in a matrix. A principal component analysis (PCA) of the fitted values produced canonical eigenvalues and a matrix of canonical eigenvectors, used to calculate the coordinates of the sites (chickens), either in the space of the original response variables, or in the space of the explanatory variables (i.e. on the fitted response variables). The residual values from the multiple regressions were also submitted to a PCA to obtain an unconstrained ordination of the residuals.

The adjusted R^2^ value was used to test whether the inclusion of explanatory variables was a significantly better fit than the null model, and a forward selection process was used to select those significant variables which explained the greatest proportion of the variance in the response data (Borcard et al., 2011). Permutation tests were used to test how many RDA axes explained a significant proportion of the variation. The Kaiser–Guttman criterion, which compares each axis to the mean of all eigenvalues, was applied to unconstrained axes, to determine those which explained variation of interest. Biplots and triplots were produced scaled according either to the distances between observations (scaling 1 in the vegan package) or the correlations between variables (scaling 2).

## 3 Results

Of the 1280 sampled, a total of 1056 birds (532 from the Horro region and 524 from the Jarso region) had complete data for all laboratory tests and all explanatory variables of interest and were included in the analysis. All birds were found to be in reasonable health at the time of sampling, with only a very few displaying any clinical signs which may have been attributable to the diseases of interest. Only 9 birds over all four seasons of testing were found to be serologically positive for NDV antibodies (HAI titre of 16 or greater); therefore this disease was not included in the ordination analyses. All other infections were present in each village and at each sampling season with the exception of IBD, where no positive birds were found in one Jarso village at any time point, and seropositive birds were detected in only three villages in Horro during the second year of sampling

Using the manufacturer's cut-off of 0.306, 3.6% of birds tested positive to IBD. Using the chosen cut-off values of 0.34, 0.61 and 0.29 for *Salmonella*, *Pasteurella* and MDV, respectively gave corresponding estimated seroprevalence of around 86%, 69% and 32% for the study samples, with only minor fluctuations between different villages and seasons. However, some birds demonstrated OD values far in excess of the positive controls, suggesting unusually high antibody titres. The parasitic diseases were also present in all villages at each time of sampling, with overall detection prevalence of 17%, 56% and 34% for Ascaridida, *Eimeria* spp. and lice, respectively. Birds showed considerable variation in the intensity of infection. Signs of hyperkeratosis were seen in 41% of birds; identification of hyperkeratosis was strongly associated with a positive identification of scaly leg mites from a skin scrape (McNemar's test, *p* < 0.01).

**Fig. 1:**
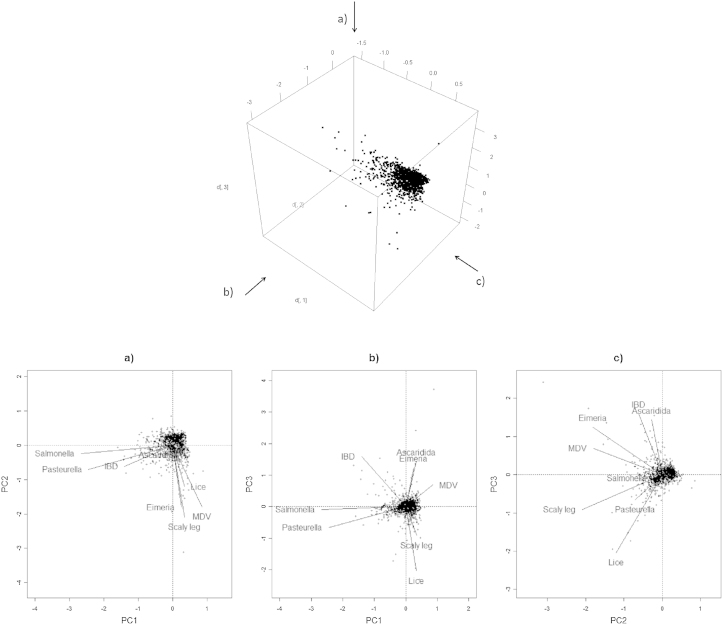
Biplots of the first three principal components (scaling 2) showing correlations between variables. Correlations are represented by the angles between the vectors rather than the apices of the lines. Labels a, b and c correspond to the three-dimensional views shown above.

After constructing the correlation matrix of responses to each infection, PCA was used to explore the variation in the scaled dataset. Regardless of the prior data transformation used, this suggested that three axes represented interesting variation in the data (using the Kaiser–Guttman criterion), and the other axes displayed essentially random variation. However, the first three axes represented less than 50% of the variation, accounting for 17.0%, 14.7% and 13.2% (in total, 44.9%), respectively on the untransformed data. Fig. 1 shows that the majority of the data points are intermediate in all variables, with a relatively small number of birds spread out by the three axes.

The first axis principally represents the variation contributed by the *Salmonella* and *Pasteurella* titres, which, as shown in the biplots, make up one group of correlated variables (Fig. 1). The majority of the second axis consists of the variation added by scaly leg mite, MDV, *Eimeria* and, to a lesser extent, lice*.* This second group of correlated variables is approximately orthogonal to the first group, indicating almost no correlation between these groups. Lice counts and IBD titre are negatively correlated and form the principal part of the variation represented by the third axis; lice have a low positive correlation with scaly leg and *Pasteurella*. Ascaridida principally contribute to the fourth (non-significant) axis, and has some correlation with *Eimeria* and IBD.

Analyses following other data transformations showed similar overall groupings, although binarisation emphasised negative correlations between scaly leg and both IBD and *Eimeria*; whilst Hellinger transformations gave more weight to the distances between the three transformed parasite counts and highlighted negative correlations between lice and *Eimeria*, and between Ascaridida and scaly leg (data not shown). All PCA's illustrated a strong correlation between *Salmonella* and *Pasteurella* which consistently contributed the 1st principal component and therefore the greatest variation in the data.

A forward selection process identified the following explanatory variables for inclusion in the RDA model: bird production status and weight; and at the household level, the use of chemical sprays for parasite control and whether the household had had any recent outbreaks of disease in their chicks or growing birds. Variables which were tested but not deemed to significantly improve model fit included the season in which the household was sampled, the village or region, body condition, age of the bird, and where the bird had come from.

**Fig. 2:**
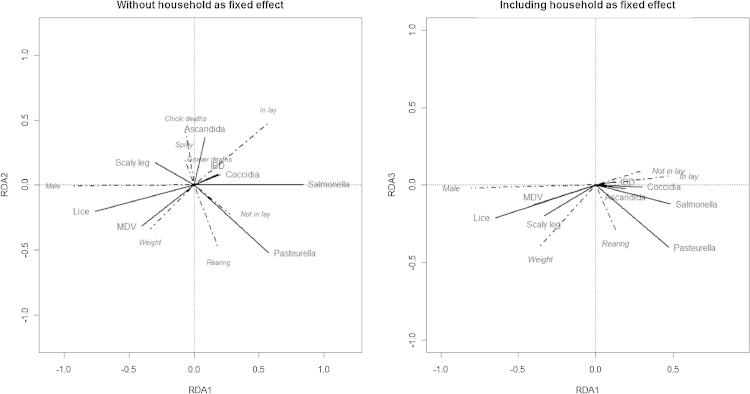
Triplots showing the relationships between response and explanatory variables between the first two canonical axes before and after incorporating household as a fixed effect in the RDA analysis. Angles between variables reflect their correlations. Solid lines represent pathogens (outcomes); dashed lines represent the explanatory variables.

Although statistically significant, the explanatory variables accounted for only a very small proportion of the total variation in the dataset (adjusted R-squared value 0.03), and a permutation test of these axes suggested that 3 of them described significant variation. However, when the household-level variation was partialled out from the model by fitting this as a fixed effect prior to fitting other explanatory variables (the permutation tests used preclude this variable from being fitted as a random effect), this explained around 59% of the variation in disease responses, and left only bird weight and production status as significant bird-level explanatory variables, and only two significant RDA axes (Fig. 2).

Some relationships were evident between variables: Males birds were positively correlated with lice infestation but negatively with *Salmonella*, and MDV was also correlated more with males and/or birds with heavier weights. Higher *Pasteurella* titres were observed in females which were either rearing chicks or not in production. Households reporting recent deaths in chicks appear to have birds with greater Ascaridida shedding, but also this variable appeared correlated with the use of household sprays to control parasites. Scaly leg was also associated with households which were using sprays in the housing to treat parasite infestations.

The residuals from the multiple regressions were also subjected to an unconstrained PCA. The Kaiser–Guttman criterion suggested 4 axes may be required to represent the residual variation of interest in the data, and the groupings of the disease variables remain much as they were before. *Salmonella* and *Pasteurella* still appear to maintain a positive correlation, even after accounting for the measured explanatory variables they have in common. Lice, MDV and scaly leg also maintain their previous cluster, although *Eimeria* became more distant from this group.

## 4 Discussion

Multiple micro- and macroparasites were circulating within the apparently healthy adult village chicken population in Ethiopia. Traditionally, epidemiology has tended to concentrate on individual diseases, but the effect of other parasites on the infection of primary interest should not be overlooked. However, identifying potential interactions between parasites and testing the strength of these associations, especially when the order of infection is unknown, remains a complex issue. The use of ordination methods was found to be a useful exploratory tool with this dataset, and appeared to be robust to the combination of different types of response data (ordinal scores, counts and serological titres), perhaps giving it wider applications within epidemiology.

PCA does not require normally distributed variables, but in community ecology count data are frequently transformed (e.g. Hellinger or chord transformations) in order to reduce the effect of large counts and to eliminate the apparent correlation of sites with double zeroes, (i.e. where two species may be absent, but for different reasons; Zuur et al., 2007). However, we did not find any advantage to using the Hellinger transformation with our parasite data. Because this transformation gives a Euclidean distance in the space of each site's (i.e. bird's) total parasite abundance, standardising the data gave a large weight to the difference between 0 and 1 parasite, but no difference to the difference between 1 and 60 parasites, if the other parasites were held constant. Since parasite intensity is important for bird health, it was decided that total burden should not be lost from the analysis. In addition, the test methods used for detecting parasite infection are not especially sensitive and the data is likely to contain a number of false negatives. The serological data, being already continuously distributed, did not require prior transformation to avoid the issue of double zeroes, and log transformations or dichotomisation to reduce the impact of outlying values resulted in relatively minor differences to the main components. In an analysis of river water quality variables, Cao et al. (1999) suggested transforming data to give weightings to observations which were outside the normal range of each variable, in order to give more biological relevance to the analysis. The untransformed scaled data maintained the distances between those birds whose infection measurements may be considered outliers from the general population. Since these may be of most interest, either in terms of their immune response and infection tolerance or because they are likely to be significant contributors to pathogen spread in this population, analysing and presenting the scaled untransformed data appeared to be of most value in this context.

The results of the ordinations showed some interesting groupings of pathogens which may warrant further investigation. These correlations were maintained even after in-common measured risk factors were accounted for, and even after accounting for the effect of farm, which might explain common exposures due to management. Therefore it would appear that either there are some attributes of the birds themselves which dictate the set of pathogens they harbour, or certain pathogens increase the risk of infection/exposure to others, resulting in the observed correlations.

The closest correlation was seen between *Salmonella* and *Pasteurella* measurements; as both of these were measured by ELISA, this may be a reflection of individual's ability to mount an adaptive immune response. The higher antibody titres were correlated with being female, and *Pasteurella* titres showed a particular correlation with females which were rearing chicks. *S. enterica* serovar Pullorum infections have been shown to recrudesce at the onset of lay in females, with the increase in bacterial numbers triggering a subsequent increase in antibody titres (Wigley et al., 2005). However, this increase in bacterial numbers does not occur in carrier males at the onset of sexual maturity. In females, sexual maturity corresponds with a decrease in T lymphocyte numbers leading to suppression of cellular immunity (Johnston et al., 2012); therefore it is plausible that the reproductive demands placed on females may increase pathogen load, not just of *Salmonella* but of other chronic or frequently encountered infections. It is also possible that there is some other unmeasured risk factor in common, such as different male and female behaviours, which increases exposure to these pathogens, and hence antibody titres. Although all birds in our study were unconfined and scavenged freely, some cocks were observed to roam for some distance, whilst hens, especially those with chicks, tend to stay close to the homestead. This may give different rates of contact with chronically infected birds, which are thought to be the major route of transmission of both *Salmonella* and *Pasteurella* infections.

The second clustering of diseases grouped MDV loosely with *Eimeria*, and MDV also showed a positive correlation with lice counts and scaly leg scores. MDV would be expected to negatively impact the immune response through lymphocyte depletion, and previous findings have demonstrated that MDV-infected birds are less able to clear *Eimeria* infections (Biggs et al., 1968). The antibody titre to MDV is indicative of exposure, but it is unclear as to how it relates to the extent of disease, as it is primarily a cellular immune response which regulates the latent phase of this viral infection. Birds with the highest titres tended to have low to moderate oocyst counts, and birds with very high oocyst shedding tended to have moderate MDV antibody titres (results not shown). However, a cellular immune response is also important in controlling *Eimeria*, and it may be that birds tolerating MDV infections are also able to moderate *Eimeria* infections to a certain extent, but not necessarily clear them as effectively as an MDV-uninfected bird. This grouping of diseases was most closely associated with male birds and/or heavier birds in the RDA. Males infected with MDV are less susceptible to the development of tumours (Payne and Venugopal, 2000), and in other avian species larger body sizes have been shown to correlate with greater abundances of lice (Møller and Rózsa, 2005); therefore it may be that males are simply tolerating these infections better, whilst females are more likely to succumb.

There was a large group of birds which were not spread out by the ordination analyses. *Salmonella* and *Pasteurella* antibody distributions were positively-skewed, with the cut-off values falling well below the mean. High antibody titres against *Salmonella* O9 serotypes may be the consequence of the establishment of a carrier state such as that found in some birds following *S.* Pullorum infection (Chappell et al., 2009); this may be the reason behind the occurrence of a low number of birds with antibody levels far in excess of our positive control. The more moderate levels of antibody titres may represent exposure to *S.* Enteritidis or *S.* Gallinarum. *P. multocida* is also capable of establishing a carrier state, although the mechanisms and the immune response to this bacterium remain largely unstudied in poultry (Wigley, 2013). This clustering of birds, which were intermediate in all antibody measurements, may therefore suggest that most of these birds have been exposed to *Salmonella* and *Pasteurella*, but are probably not chronically infected, and are negative for IBD and MDV and all parasites. This group of birds is challenging to interpret; low antibody titres may mean that birds have not been exposed, were infected too long ago to detect the response or simply have a poor immune response, possibly as a result of another immunosuppressive or immune-modifying infection. For the parasite species that we measured more directly, a negative count may again mean a bird has not been exposed, or has been previously infected and cleared the infection.

As illustrated by the number of axes required to explain the residual variation in disease responses, there were almost no birds which had high responses/counts to more than one pathogen group. This may in part be due to other opposing and mutually exclusive risk factors, such as sex, in that higher lice burdens are associated with males, but females tend to have higher *Salmonella* titres. Whilst differences between villages were minor (reflected in the non-significant improvement of the RDA model by inclusion of the village or region variables), there appears to be considerable within-village variation. Although including household as a partial fixed effect in the RDA then prohibited the inclusion of variables measured at the household or village level, it did illustrate the significant contribution of household to many of the disease responses, suggesting that either there are some important, unmeasured, household factors or that exposure to these diseases at any single time point varies significantly even within a village. This may be due to geographical and management factors, but may also be due to the relatedness of birds in the same household giving them similar immune characteristics. The scarcity of co-positive birds may be due to a lack of synchronicity between infections. It is also possible that birds recently infected with two pathogens in a short time period, where we may expect both a high antibody titre and a high parasite burden, frequently do not survive to be measured, hence the lack of these in this dataset. In order to investigate many of these hypotheses, longitudinal studies are required; however, the ordination analyses has proved useful in the generation of these hypotheses, and in illustrating some potential associations, both positive and negative, between different types of pathogens, even with very different types of measurement used.

The impact of these multiple infections in the village chicken population is likely to be of greatest significance within the young stock that families rear for replacements, sale or consumption, and which generally mix freely with the adult population. The observed correlation between birds with high Ascaridida counts and households which had had recent deaths in their chicks may suggest that nematodes are of particular significance for this age group, allowing targeted interventions to be considered. However, any of these infections may also potentially impact the adult stock at times of climactic, nutritional or other stress. The results of this study have implications for interventions to improve chicken health and production in Ethiopia. For example, programmes aiming for genetic improvement of indigenous stock by selective breeding (Dana et al., 2011) need to be aware of the range of infectious diseases to which village chickens are exposed, and be careful not to lose important protective immune traits from the population. Furthermore, as demonstrated in other systems (Nacher, 2011), interventions such as de-worming could, whilst controlling for one pathogen, have implications for the infection biology and epidemiology of others; therefore any intervention undertaken in this population should carefully monitor any effects on other diseases, especially those with zoonotic implications.

## Conflict of interest statement

The authors declare they have no competing interests.
